# Use of Tocilizumab May Avoid the Need of Invasive Ventilation

**DOI:** 10.7759/cureus.17822

**Published:** 2021-09-08

**Authors:** Anurag Adhikari, Ayusha Poudel, Oshna Pandey, Barun B Aryal, Bibek Dhungana

**Affiliations:** 1 Intensive Care Unit, Nepal Korea Friendship Municipality Hospital, Madhyapur Thimi, NPL; 2 School of Medicine, Patan Academy of Health Sciences, Kathmandu, NPL; 3 Emergency Medicine, BP Smriti Hospital, Kathmandu, NPL; 4 Internal Medicine, KIST Medical College, Lalitpur, NPL

**Keywords:** covid-19, sars-cov-2, cytokines, tocilizumab, ventilation

## Abstract

Coronavirus disease 2019 (COVID-19) is an illness caused by severe acute respiratory syndrome coronavirus 2 (SARS-CoV-2). Increased pro-inflammatory cytokines including interleukin 6 (IL-6) are associated with severe forms of illnesses. The severe cases of COVID-19 require a high amount of oxygen supplementation and might even require endotracheal intubation with ventilator support. A blockade of inflammatory cascade with the use of tocilizumab has been shown to decrease the need for intubation and ventilator requirement.

## Introduction

Coronavirus disease 2019 (COVID-19) is a disease condition caused by the severe acute respiratory syndrome coronavirus 2 (SARS-CoV-2). It mainly affects the respiratory system. In the early phases of the COVID-19 pandemic, it was found that severe forms of SARS-CoV-2 infection were associated with dysregulated immune response and cytokine storm leading to inflammation of the lungs [1]. Interleukin 6 (IL-6) is a pro-inflammatory cytokine [2]. It has been hypothesized that pro-inflammatory cytokines like IL-6 play a role in severe illness due to SARS-CoV-2 [3].

Tocilizumab is one of the earliest marketed drugs that targets and blocks the interleukin-6 receptor [4]. The use of tocilizumab seemed to improve the outcome in hospitalized patients in the early phase of the pandemic [5]. We present a case of a 59-year-old man who presented with severe bilateral COVID-19 pneumonia and was deteriorated to the point where endotracheal intubation was contemplated. However, the use of tocilizumab resulted in recovery, and oxygen supplementation could be weaned gradually.

## Case presentation

**Figure 1:**
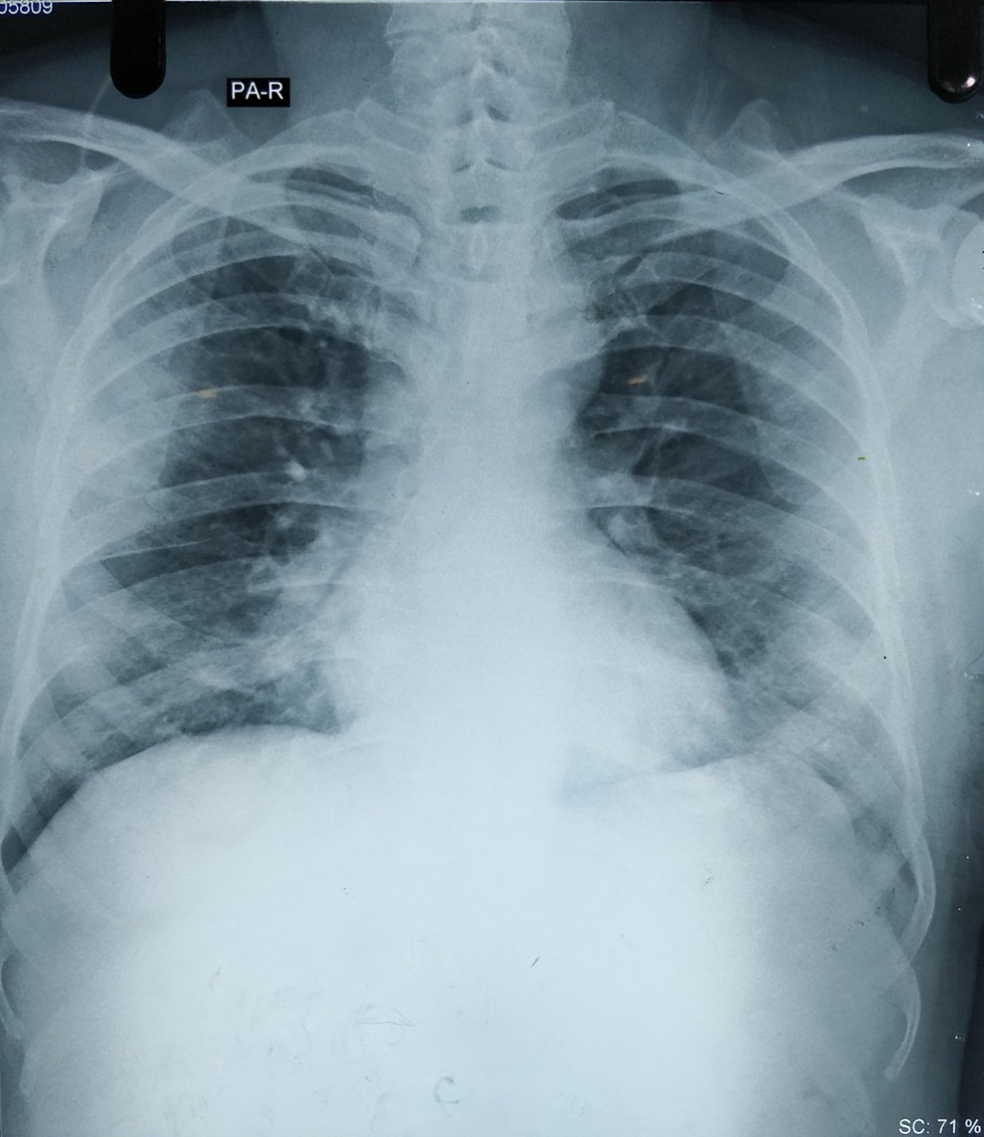
Chest X-ray demonstrating bilateral pneumonia.

A 53-year-old male was referred to our center with complaints of shortness of breath and fever for three days. A reverse transcription-polymerase chain reaction (RT-PCR) for COVID-19 was performed, which yielded a positive result three days back. His co-morbidity was hypertension for which he was under amlodipine 10 milligrams (mg) orally daily. On presentation, he was neurologically intact and afebrile. However, there was decreased air entry in the bilateral hemithoraces with bilateral basilar crepitation. He was under oxygen supplementation at 15 liters per minute (L/min) via a face mask with a reservoir bag and his oxygen saturation was maintained at 95%. His D-dimer levels were 230 nanogram per milliliter (ng/mL) (biological reference interval 0 to 500 ng/mL). His metabolic panel was within the normal limits. His chest X-ray revealed bilateral infiltrates in the lungs and he was admitted to the intensive care unit with a diagnosis of bilateral COVID-19 pneumonia (Figure 1). He was started on intravenous piperacillin/tazobactam 4.5 grams (gm) thrice daily, intravenous vancomycin 1 gm twice daily, intravenous dexamethasone 6 mg once daily, and subcutaneous enoxaparin 60 mg once daily.

On the subsequent day, his oxygen saturation began to drop and fluctuated between 93% and 95%. He began to further deteriorate and his oxygen saturation started to fluctuate around 83% to 86%. The arterial blood gas (ABG) analysis revealed a potential of hydrogen (pH) of 7.434, partial pressure of arterial oxygen (PaO_2_) of 65 millimeters of mercury (mmHg), partial pressure of arterial carbon dioxide (PaCO_2_) of 29.8 mmHg, and bicarbonate level of 21.4 milliequivalents per liter (mEq/L). He was then initiated on a high-flow nasal cannula (HFNC) with fractional oxygen (FiO_2_) of 90% and a flow rate of 70 L/min.

His condition got worse by the day and on the third day of admission, his peripheral oxygen saturation hovered from 81% to 85% when placed on HFNC. His ABG reports revealed pH of 7.410, PaO_2_ of 91 mmHg, PaCO_2_ of 28.8 mmHg, and bicarbonate of 19.1 mEq/L. Consideration for the need for endotracheal intubation was made; however, the patient and his relatives were reluctant to provide consent until other therapeutic options were available. The HFNC was supplemented with a reservoir bag with oxygen at 15 L/min over the cannula to maintain the oxygen saturation over 90% overnight. His procalcitonin levels were found to be 0.25 nanogram per milliliter (ng/mL) (biological reference range of 0 to 0.5 ng/mL) and the interleukin 6 (IL-6) levels were 11.9 picograms per milliliter (pg/mL) (biological reference range of less than 7 pg/mL).

On the fourth day of admission, non-invasive ventilation was trialed but the patient could not tolerate it. The patient was then kept in a prone position with oxygen supplementation via HFNC at FiO_2_ of 80% and flow rate of 50 L/min in addition to a face mask with a reservoir bag providing oxygen at 10 L/min. A single dose of tocilizumab 400 milligrams (mg) was given intravenously to the patient on the same day after being diluted in 100 milliliters (mL) of 0.9% normal saline over one hour.

Interestingly, the patient started getting better overnight and his oxygen saturation was maintained with HFNC alone. A trial of oxygen supplementation at FiO_2_ of 60% with a venturi mask was done but he was unable to maintain the oxygen saturation. The ABG analysis showed pH of 7.44, PaO_2_ of 60 mmHg, PaCO_2_ of 27.9 mmHg, and bicarbonate of 20 mEq/L.

**Figure 2:**
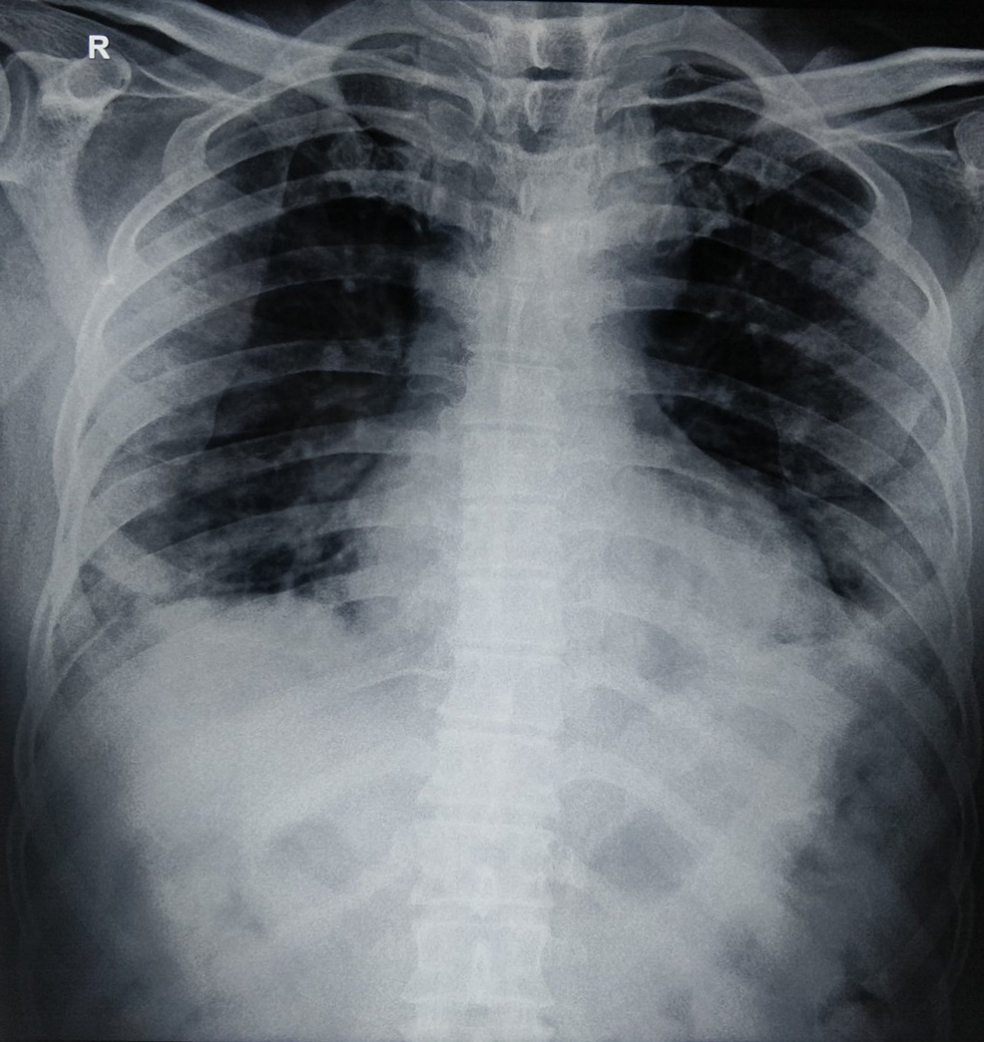
Chest X-ray after two days of administration of tocilizumab.

On the fifth day of admission, he showed significant improvement. He was able to maintain peripheral oxygen saturation above 92% with oxygen supplementation via venturi mask of 60% when supine and above 64% when prone. The ABG analysis revealed pH of 7.42, PaO_2_ of 80 mmHg, PaCO_2_ of 28 mmHg, and bicarbonate of 18 mEq/L. His chest X-ray also showed improvement (Figure 2).

On the following two days, the oxygen supplementation was gradually tapered from FiO_2_ of 60% to 31% via venturi mask. On the 13th day of admission, he was transferred to the ward with oxygen supplementation of 5 L/min via nasal prongs.

His laboratory studies during the course of treatment are summarized in Table 1.

**Table 1:** Laboratory studies of the patient. cells/mm^3^: cells per cubic milliliter; gm/dL: gram per deciliter; mg/dL: milligram per deciliter; mEq/L: milliequivalent per liter.

Laboratory parameters	At admission	On-shift to ward	Reference range
Total leucocyte count	9,600	14,200	4,000-11,000 cells/mm^3^
Neutrophils	93	91	40%-75%
Lymphocytes	6	5	20%-50%
Eosinophils	0	1	1%-6%
Monocytes	1	3	2%-10%
Basophils	0	0	0%-1%
Hemoglobin	12.9	13.2	13-18 gm/dL
Platelets	110,000	291,000	150,000-400,000 cells/mm^3^
Urea	23.4	26	15-45 mg/dL
Creatinine	1.35	1.3	0.4-1.4 mg/dL
Sodium	139	139	135-146 mEq/L
Potassium	4.2	4.1	3.5-5.3 mEq/L

## Discussion

The severe forms of COVID-19 illnesses have a poor prognosis. The severity of illness with SARS-CoV-2 is found to be correlated with the pro-inflammatory cytokine levels in the plasma [6]. Severe hospitalized patients in a study had an increased level of interleukin 6 (IL-6) [7]. Tocilizumab is a monoclonal antibody against IL-6 and its use had been well demonstrated for rheumatoid arthritis prior to the pandemic [4]. The role of cytokine storm in severe SARS-CoV-2 infection and elevated IL-6 levels led to the use of tocilizumab for COVID-19 illness. The use of tocilizumab showed benefit in hospitalized COVID-19 patients in China [5]. The subsequent observational study proved that the use of tocilizumab had a mortality benefit in critically ill patients [8]. The Randomized, Embedded, Multifactorial Adaptive Platform Trial for Community-Acquired Pneumonia (REMAP-CAP) demonstrated that tocilizumab had improved outcomes including survival [9].

Randomized controlled trials, however, have not uniformly proven the therapeutic benefit of tocilizumab in COVID-19 patients. A randomized controlled study demonstrated that it reduced the need for progression to mechanical ventilation but had no effect on survival [10]. However, another study showed neither benefit in preventing intubation nor in death for moderately severe hospitalized COVID-19 patients [11]. Another randomized trial in COVID-19 pneumonia patients with PaO_2_/FiO_2_ ratio between 200 and 300 mmHg, revealed that tocilizumab had no benefit on disease progression [12].

Consistent with the majority of randomized trials, meta-analyses have been unable to suggest the use of tocilizumab beyond doubt. A similar study could not find the evidence for the use of tocilizumab and recommended the halting of its use until further evidence was available [13]. Another systematic review found the evidence insufficient and advised the use of tocilizumab as an experiment [14]. The United States Food and Drug Administration (FDA) has recently approved tocilizumab for hospitalized patients of age two years and more, who are also receiving corticosteroids and supplemental oxygen in any form [15].

However, Randomised Evaluation of COVID-19 Therapy (RECOVERY) trial has revealed that tocilizumab resulted in improvement of survival as well as other clinical outcomes in hospitalized COVID-19 patients with hypoxia regardless of respiratory support [16]. More studies might be needed to elucidate the actual role of tocilizumab.

Our case was a 59-year-old hypertensive man who presented to our institution with high oxygen requirement. He had bilateral pneumonia and elevated interleukin levels. With time, his condition started deteriorating and his oxygen requirement progressively increased. In view of increased oxygen requirement, a high-flow nasal cannula (HFNC) was used initially. As the patient was unable to maintain peripheral oxygen saturation with HFNC, there was an imminent need for endotracheal intubation. However, a single intravenous use of 400 mg of tocilizumab decreased the oxygen requirement, and the need for intubation was avoided. A similar case was described in the United Kingdom where the use of tocilizumab avoided the need for intubation [17].

## Conclusions

The severity of COVID-19 is associated with the increased levels of pro-inflammatory cytokines including IL-6. Blockade of the inflammatory cascade has led to better outcomes in severe cases of COVID-19. Use of tocilizumab early in the course of illness might lead to improvement of the patient and avoid the need for endotracheal intubation.
